# Harnessing the Natural Biology of Adeno-Associated Virus to Enhance the Efficacy of Cancer Gene Therapy

**DOI:** 10.3390/v13071205

**Published:** 2021-06-23

**Authors:** Jacquelyn J. Bower, Liujiang Song, Prabhakar Bastola, Matthew L. Hirsch

**Affiliations:** 1 Lineberger Comprehensive Cancer Center, University of North Carolina at Chapel Hill, Chapel Hill, NC 27514, USA; 2 Department of Ophthalmology, University of North Carolina at Chapel Hill, Chapel Hill, NC 27599, USA; liujiang@email.unc.edu (L.S.); pbastola@email.unc.edu (P.B.); 3 Gene Therapy Center, University of North Carolina at Chapel Hill, Chapel Hill, NC 27599, USA

**Keywords:** adeno-associated virus, AAV, cancer gene therapy

## Abstract

Adeno-associated virus (AAV) was first characterized as small “defective” contaminant particles in a simian adenovirus preparation in 1965. Since then, a recombinant platform of AAV (rAAV) has become one of the leading candidates for gene therapy applications resulting in two FDA-approved treatments for rare monogenic diseases and many more currently in various phases of the pharmaceutical development pipeline. Herein, we summarize rAAV approaches for the treatment of diverse types of cancers and highlight the natural anti-oncogenic effects of wild-type AAV (wtAAV), including interactions with the cellular host machinery, that are of relevance to enhance current treatment strategies for cancer.

## 1. Adeno-Associated Virus: Discovery and Biology

**Figure 1:**
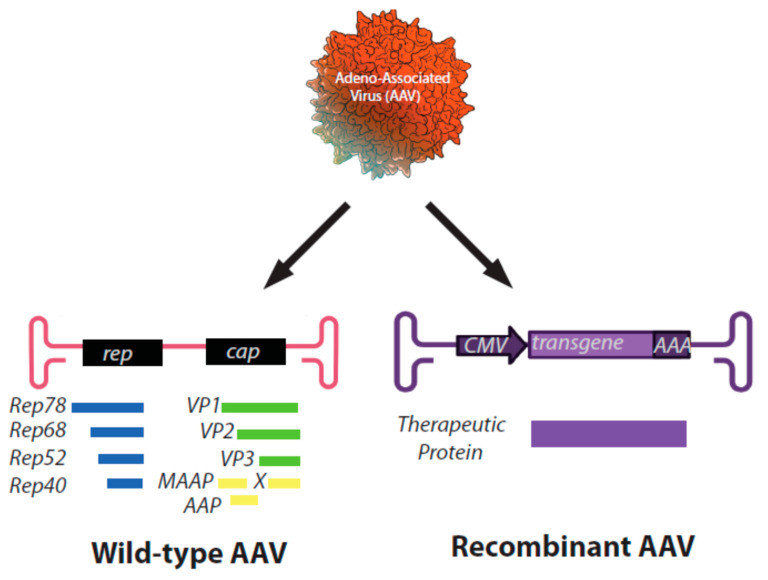
Schematic representation of genomes of a wild-type AAV virus (left) and a recombinant AAV particle (right). Proteins encoded by each ORF are listed below the appropriate gene.

Adeno-associated virus (AAV), a member of the *parvoviridae* family, was first discovered as a defective contaminant virus of an adenovirus preparation because it required a helper virus to replicate. It was largely thought to be non-pathogenic as no adverse events were noted upon infection in cell culture or in several mammalian species inoculated with the virus [1]. AAV harbors a single-stranded DNA genome of approximately 4.7 Kb that contains two primary open reading frames (ORFs) (Figure 1). Reports have shown that the first ORF, *rep,* encodes four versions of the replication protein (Rep78, Rep68, Rep52, and Rep40), and the large Rep proteins (78/68) maintain site-specific DNA binding and endonucleolytic activity conferred by approximately the first 200 N-terminal amino acids. The smaller Rep proteins maintain the helicase activity and are thought to assemble as a multimeric complex to elicit replication. The second ORF, *cap,* encodes three isoforms of the capsid protein (VP1, VP2, and VP3). The assembly activating protein (AAP) is encoded through an alternative ORF within the *cap* gene and is reported to aid in genome packaging of several capsid serotypes [2,3]. Two additional proteins encoded by *cap*, protein X and the membrane associated accessory protein (MAAP), have been described [4,5,6]. Although their precise functions in AAV biology are to date unclear, these proteins have been suggested to be involved in the enhancement of AAV replication and virion egress, respectively.

The AAV genome is terminated by inverted repeat DNA sequences (Inverted Terminal Repeats; ITRs) that are required for replication, capsid packaging, and long-term intracellular persistence [7]. To generate recombinant AAV (rAAV) for therapeutic applications, the AAV coding region is removed, and a transgenic sequence is positioned between the flanking ITRs. The transgenic sequence is then replicated and packaged into an AAV capsid via the expression of the wtAAV genome in trans and without ITRs, in the presence of AAV “helper” plasmids which influence wtAAV gene expression. The resultant particles are a protein capsid, with ITRs flanking the genetic sequence of interest (<5 kb), on a single strand of DNA of either polarity [7,8].

AAV requires a helper virus for replication such as adenovirus or herpes simplex virus in a process that is not completely understood [1,9]. The large Rep proteins (68/78) are capable of binding DNA including a defined 16-nucleotide rep binding element (RBE) located within the AAV ITR. Rep binds to the RBE as a multimeric complex and is reported to elicit site- and strand-specific endonuclease, DNA/DNA helicase, RNA/DNA helicase, and ATPase activities [10]. In addition to these roles, Rep can also self-regulate the activity of its own p5 promoter in an orchestrated sequence related to external helper functions and to its own abundance [11,12]. Interestingly, Rep78 has also been shown to regulate non-native gene expression of other viruses and multiple human genes. For example, a purified Rep78-maltose binding protein (MBP) fusion was shown to bind and repress gene expression from the HIV-1 LTR [13]. Other studies have shown that Rep78 can bind to the human promoter sequences of multiple oncogenes, including *c-fos*, *c-myc*, and *H-ras*, and downregulate reporter gene activity [14,15]. Consistently, RBEs or RBE-like sequences have been identified in multiple locations of the human genome, other viral genomes, and other animal genomes: in fact, a bioinformatics search for the consensus 16-mer core Rep recognition sequence has also been found in or flanking multiple human genes, several of which play roles in DNA repair and cell cycle arrest including BRCA1, ERCC1, and GADD45 [16]. Although these binding sites were confirmed using a Rep68-MBP purified fusion protein in electrophoretic mobility shift assays, the endonuclease activity of Rep68 or effects on human gene expression were not reported. Thus, it is unclear whether Rep68 might mediate AAV genome integration and/or affect gene regulation at these loci.

The *cap* gene encodes three structural proteins: VP1, VP2, and VP3. These proteins assemble in a 1:1:10 ratio, respectively, to generate a relatively simple icosahedral capsid structure. The capsid proteins determine the serotype of the resulting AAV particle and influence its transduction efficiency at multiple discrete steps of the infection pathway. Once AAV enters the cell via receptor-mediated endocytosis, conformational changes of the capsid facilitate endosomal escape, nuclear trafficking, and entry, where it is thought to uncoat partially and/or completely, releasing the ssDNA genome [3,17]. The host cellular DNA replication machinery subsequently synthesizes the complementary sequence presumably via leading strand synthesis initiated by the 3′ ITR, termed “second-strand synthesis”, and creating a single self-complementary DNA strand capable of duplex formation via self-annealing [18,19]. Depending on the abundance of nuclear ssDNA genomes, opposite polarity strand annealing also generates double-stranded AAV (dsAAV) DNA. Once the AAV genome assumes a double-stranded form, the genes encoded are competent for transcription regardless of whether the virus contained a wild-type genome or a transgenic sequence. Often, the AAV genome will undergo intra- or inter-molecular circularization, a process facilitated by the recombinogenic nature of the ITRs that is postulated to form larger concatemers as its persistent episomal form.

In addition to the Rep and Cap proteins, wtAAV relies on other exogenous proteins to complete its life cycle. Although wtAAV was discovered in the presence of an adenovirus, several other viruses can provide helper function for wtAAV replication, such as Herpes Simplex Virus and Human Papillomavirus [20,21]. It has even been suggested that wtAAV is not strictly a “defective” virus, as autonomous replication in particular contexts, such as in skin cells, has been reported [21]. Other biological effects of AAV, such as transduction efficiency, can also be enhanced by the presence of a helper virus (such as adenovirus) or by exposure to small molecule inhibitors [18,22]. Interestingly, Nicolson et al. showed that five different categories of small molecules could enhance rAAV transduction 2–200 fold including topoisomerase II poisons, DNA damaging agents, epigenetic modifiers such as HDAC inhibitors, DNA intercalators, and proteosome inhibitors [22]. These findings are relevant for all rAAV therapeutic applications; however, many of these drugs are currently used as chemotherapeutics for multiple types of cancer, suggesting that a well-designed combination of chemotherapy and AAV-based gene therapy approaches could produce a synergistic effect to enhance cancer cell death.

## 2. Overview of Cancer Gene Therapy Approaches with rAAV

The rAAV vectors, unlike wtAAV, contain only the flanking ITR regions of the viral sequence. To prepare a rAAV vector for gene therapy applications, portions of a helper virus are provided on a plasmid that facilitate replication of the rAAV, but cannot produce helper virus to prevent contamination of the therapeutic rAAV with other viral particles. The use of rAAV vectors for gene therapy boasts several advantages, including, but not limited to: (1) no known pathogenesis associated with AAV infection, (2) the ability to deliver genetic material to dividing and non-dividing cells, (3) conferring long-term transgene expression, and (4) a favorable safety profile. The FDA has approved AAV-based gene therapies for the treatment of the rare genetic diseases Leber’s congenital amaurosis (Luxturna^®^) and spinal muscular atrophy (Zolgensma^®^), solidifying the role of rAAV therapeutic approaches for monogenetic diseases. Thus, it is not surprising that similar or related therapeutic strategies have been evaluated for multigenic diseases such as cancer. In fact, a multitude of rAAV approaches for cancer gene therapy are under examination in pre-clinical models. Curiously, rAAV clinical trials for cancer gene therapy applications to date have been relatively sparse when compared to the number of adenovirus-based cancer gene therapy trials (23 trials for rAAV vs. 436 trials for adenovirus) and are heavily focused on the use of rAAV to express the GM-CSF cytokine to induce an immune response against prostate cancer cells (Table 1) [23].

Due to the heterogeneous nature of cancer cells both among and within individual tumors, pre-clinical rAAV cancer gene therapy approaches have widely varied in an effort to take advantage of an assortment of cancer-driving cell signaling networks. For example, several groups have attempted to overexpress tumor suppressors and/or DNA repair genes, such as p53, to enhance apoptosis in a variety of cell line and xenograft models including breast cancer and cervical cancer models [24,25]. Another group has expressed the c-terminal portion of hTERT in AAV vectors to induce telomere dysfunction in a xenograft mouse model of glioblastoma [26]. Others have overexpressed endogenous anti-angiogenic factors such as endostatin or angiostatin in rAAV to reduce new blood vessel formation in tumors including melanoma, lung, and pancreatic cancer models in the hopes of reducing metastatic spread [27,28]. rAAV vectors expressing “suicide” genes such as HSV-TK in combination with ganciclovir have also been used to enhance death in breast cancer cell lines [29,30]. Finally, rAAV vectors encoding monoclonal antibodies such as bevacizumab to target VEGF and prevent angiogenesis have also been encoded in AAV vectors and reduce tumor burden in a xenograft mouse model of ovarian cancer [31]. Unfortunately, such gene therapy approaches targeting individual signaling pathways may be difficult to implement from a translational/commercial perspective, due to the inherent diversity of tumors. For example, there are at least five independent subtypes of breast cancer that differ in their presentation, molecular pathology, and response to chemotherapy [32,33]. Although a portion of each tumor subtype contain known cell signaling defects in a single pathway, such as the BRCA1 pathway, it is not feasible to identify, test, develop, and produce a rAAV vector targeting each individual component of the mutant BRCA1 signaling cascade. In addition, such an approach would require the development of high-level clinical diagnostics to determine the defective molecular pathway for each patient. Furthermore, many of the proteins involved in these DNA repair pathways are quite large; thus, not all components of these pathways are targetable due to the 4.8-Kb packaging limitation for rAAV.

Other notable approaches using rAAV include the delivery of sequences that can alter/regulate gene expression specifically in cancer cells. These approaches include the expression of shRNA(s) that specifically target oncogenic driver genes such as FHL2 in a colorectal cancer model [34]. Another pre-clinical study using a cervical cancer xenograft mouse model was treated with rAAV2 expressing an shRNA to deplete the HPV-E6 protein; this approach reduced the tumor burden to non-detectable levels [35]. Alternate approaches expressing microRNAs (miRs) such as miR-26a specifically induced apoptosis in liver tumor cells in a genetically engineered mouse model [36]. Promoter restriction has also been used to specifically target cancer cells, such as the use of the CXCR4 promoter, which is overexpressed in breast cancer, and could be combined with the aforementioned “suicide gene therapy” approach [37]. From a feasibility standpoint, such approaches bypass some of the translational concerns including the size limitation of rAAV packaging as the genetic components are much smaller than full-length proteins and the need for an individual rAAV for each aberrant signaling protein, because it can be engineered to harbor multiple shRNAs/miRs. Furthermore, shRNA and/or miRs expressed in rAAV may be an excellent alternative to currently employed small molecule inhibitors, which often initially decrease the tumor burden of patients, but almost always ultimately result in acquired resistance. rAAV’s ability to provide long-term genetic expression of multiple shRNA/miR cassettes may be able to overcome this challenge by expressing multiple cassettes and potentially decreasing targeted therapy resistance. However, they would still require advanced molecular diagnostic assays for each individual patient, which could impede clinical applications.

Another approach to rAAV cancer gene therapy has been to modify capsid proteins via rational design/directed evolution to enhance/restrict rAAV transduction of cancer cells. Several groups have attempted to rationally design capsid proteins fused to a ligand that binds a specific receptor on cancer cells, such as the designed ankyrin repeat proteins [38]. Others have endeavored to deliver rAAV specifically to increase transduction of dendritic cells to induce a T cell-mediated immune response to the cancer [39]. Additional studies have generated “locked” versions of AAV capsids that contain matrix metalloproteinase (MMP) cleavage sites to promote capsid uncoating and, therefore, transgene expression specifically in cancer cells that overexpress MMPs, which are thought to contribute to their metastatic behavior [40]. Directed evolution approaches that enhance transduction of glioma cells when compared to natural AAV capsid serotypes have also shown promise for previously difficult to transduce brain tumor cells [41]. Such approaches are expected to provide enhanced delivery of transgenes, and in combination with the aforementioned molecular strategies, have the potential to increase rAAV’s efficacy through improved cancer cell specificity.

One of the most popular approaches for cancer gene therapy is to express modulators of the immune system from rAAV to generate an immune response to a particular cancer. For example, several groups have attempted to deliver immune stimulating cytokines via rAAV to induce a tumor-specific host immune response. Ma et al. have shown in a pre-clinical model that rAAV expressing soluble TRAIL can induce apoptosis in liver metastases via hepatic portal vein injection in a mouse xenograft model of lymphoma, suggesting that rAAV–TRAIL may be able to treat liver metastases arising from hematogenous metastasis [42]. Another immune based approach is to stimulate antigen presenting cells (APCs) with rAAV-encoded antigens such as carcinoembryonic antigen (CEA) as a vaccine for colon cancer; indeed, this approach was shown to reduce the development of colon tumors in mice [43]. Many of these studies demonstrated significant or complete tumor regression in multiple in vivo mouse model systems; however, clinical translation of cancer gene therapies remains on the horizon. Typically, clinical translation of cancer therapies from mice have been historically difficult; thus, it remains to be seen whether any of these approaches will have efficacy in the human context.

## 3. WT AAV Induces Cancer Cell Death

Although most approaches to cancer gene therapy have utilized rAAV strategies, wtAAV itself is reported to have inherent anti-tumorigenic properties that specifically disrupt cancer cell physiology. Much of the early work in this area was generated in the last century and focused on the ability of AAV to prevent viral transformation of mammalian cells upon co-infection with oncogenic viruses such as herpesvirus, bovine papillomavirus, and human papillomavirus [44,45,46,47]. In 1981, De la Maza and Carter showed that AAV2 particles harboring sub-genomic pieces of AAV2 DNA (known as defective interfering AAV particles or DI particles), presumably containing the ITR, suppressed Adenovirus-12-induced tumorigenesis in a newborn Syrian golden hamster model [48]. Subsequently, Khleif et al. used an adenovirus derived E1a/ras oncogene plasmid to induce transformation in mouse NIH/3T3 cells [49]. Co-transfection of a non-viral wild-type AAV2 plasmid almost completely suppressed transformation. After examining plasmids with either a defective *rep* or *cap* gene, it was determined that the *rep* mutant plasmid failed to suppress E1a/ras transformation. In contrast, a *cap* mutant plasmid or a plasmid that contained no viral origin of replication suppressed transformation as well as wt AAV2. *rep* mutant plasmids that expressed only Reps 52/40, spliced Reps 68/40, or a K446H mutant located in the purine binding site showed transformation suppression of varying levels. A plasmid containing ITRs, but no *rep* or *cap* sequences, did not suppress transformation, suggesting that the rep protein was required for suppressing the transformation of mouse NIH/3T3 fibroblasts by the adenovirus E1a and ras proteins, at least in a plasmid context [49]. Although the mechanism was not explored in this study, it was subsequently shown that Rep78 binds to p53 and prevents its degradation by Adenovirus, suggesting that p53 may play a role in wtAAV’s capacity to reduce cellular transformation induced by helper viruses [50].

Later reports have demonstrated that wtAAV can directly induce cell death in multiple types of cancer cell lines. Furthermore, the toxicity of wtAAV appears to be specific to cancer cells, as it does not seem to induce cell death in most primary or diploid cell lines. For example, more recent work has suggested that wtAAV2 can induce death in HPV-infected cells, while HPV negative keratinocytes remain unaffected [51]. AAV2 appeared to induce apoptosis that correlated with the expression of several of the Rep isoforms (78, 68, and 40), whereas Rep expression was not observed in the HPV negative keratinocytes. AAV replication was detected in both HPV-infected cervical cancer cells and keratinocytes, albeit at slightly weaker levels in the HPV negative keratinocytes. AAV2 infection of the cervical cancer cells increased the number of cells in S phase, the number of cells with subG1 DNA content, CDK1 kinase activity, and active pRB, when compared to non-infected cells [51]. Furthermore, a reduction of p21/p16/p27 proteins normally associated with a G1 arrest was also reported. Taken together, these data suggest that the HPV-infected cells bypassed the G1 cell cycle checkpoint and facilitate entry into S phase, perhaps linking the observed cell death to Rep and/or wtAAV genome replication in the context of HPV-transformed cells.

wtAAV’s tumoricidal effects are not limited to HPV-transformed cells. In fact, similar results were seen in multiple types of breast cancer cell lines including hormone receptor positive and triple negative breast cancer cell lines, suggesting that wtAAV exhibits a broader anti-tumorigenic effect that is not specific to a single cell line or class of cell lines [52]. Again, cell death correlated with Rep expression, AAV replication, and a higher percentage of cancer cells in S phase; however, the cell death occurred through both caspase-dependent and caspase-independent mechanisms among the cell lines, suggesting that AAV is capable of activating multiple cell death signaling mechanisms that can override survival signals inherent to tumor cells [52]. Finally, wtAAV2 infection was concurrent with Rep 78/52 expression and viral replication in a breast cancer cell line xenograft model, and tumor growth was retarded [53]. Because the Rep78 protein is reported to induce apoptosis via caspase 3 activation independently of p53, wtAAV may exhibit a broader range of tumoricidal activity for multiple types of cancers, regardless of p53 status [54].

Although the above studies demonstrate that Rep protein levels and viral replication are correlated with the tumoricidal effects of wtAAV, the mechanism(s) is/are not well defined. The Rep78 protein is thought to induce a prolonged S phase arrest via multiple mechanisms [55]. Rep78 binds to the Cdc25A phosphatase and prevents it from interacting with the cyclin-dependent kinases, Cdk2 and Cdk1, which are required for S phase entry and mitotic entry, respectively [55]. Both large Rep proteins (78/68) can also nick the chromatin and induce an ATM-dependent arrest leading to G1 and G2 arrest [55]. Additional requirements for the sustained S phase arrest appear to include hypophosphorylated pRB, the zinc finger domain of Rep78, and Rep78 endonuclease activity [55,56]. Taken together, these data suggest that wtAAV can stall cells in S phase, presumably for the purposes of enhancing its ability to undergo second-strand synthesis [57]. Because most cancer cells exhibit functional defects in one or more cell cycle checkpoints and wtAAV is considered non-pathogenic, it may be possible to enhance the tumoricidal activity of wt and/or rAAV by exploiting its natural life cycle to deliver transgenes that exhibit increased toxicity in the S phase of the cell cycle. Upon a wtAAV-induced DNA damage response, cancer cells are perhaps likely to be overwhelmed by the high levels of induced “DNA damage” signaling elicited upon AAV infection and forced to undergo apoptosis and/or necrosis. As non-tumorigenic cells generally have a higher capacity to repair damaged DNA and can maintain a cell cycle arrest until that damage is repaired, they are predicted to be less susceptible to cell death upon wtAAV infection.

In addition to the anti-tumor effects of the Rep proteins, it has also been suggested that the AAV ITRs are capable of inducing death in tumor cells lacking p53 both in vitro and in vivo. Raj et al. have demonstrated that p53-negative tumor incidence and tumor growth were decreased in response to UV-irradiated AAV infection in a mouse xenograft model, suggesting that Rep expression is not required for the tumoricidal activity of wtAAV in cells deficient for p53 [58]. Further experimentation showed that the short hairpin ITR sequences alone were sufficient to induce death in these p53-deficient cell lines, suggesting that a second genetic feature of wtAAV may enhance its tumoricidal effects. It is likely that the inherent sequence/structure of the hairpin ITRs, which elicit a cellular DNA damage response, are difficult for cancer cells to process and thus enhance the activation of additional apoptotic and necrotic cell death mechanisms, perhaps following a replicative catastrophe [59].

Consistently, several epidemiological studies have suggested that natural AAV infection measured via seropositivity is inversely associated with cervical cancer development [60,61,62]. Other studies have shown that 50–80% of all cervical tissue samples collected for routine Pap smears in women without cervical cancer contained AAV, offering incidental human data that AAV infection may be preventative for cervical carcinogenesis [63,64]. However, it should be noted that wtAAV is capable of integrating into the host genome, particularly at the AAVS1 site on human chromosome 19 [65]. Recently, whole genome sequencing data were mined from approximately 1400 liver biopsy samples, and although the authors observed wtAAV integration events in liver tissues, they were less frequent in malignant or benign tumors (8%) than in non-tumor liver tissue (18%), with a few instances of wtAAV insertions flanking or inserted into potential oncogenes (~1.2%) [66]. AAV integration events were also observed in a long-term study of a canine cohort receiving rAAV-based gene therapy for hemophilia; however, none of the dogs showed signs of liver disease including tumors [67]. Additional studies in humans revealed no difference between the presence of AAV in multiple tumor tissues when compared to the adjacent non-tumor tissue in more than 400 cancer patients [68], and functional p53 has been shown to reduce AAV integration events [69]. Taken together, these data suggest that although integration of wtAAV is possible, it likely is not a cause of tumorigenesis; rather, its integration and amplification are a consequence of the altered biology of cancer cells. Although the mechanism is not yet clear, potential factors may include alterations in chromatin condensation, increased cell proliferation, and loss of tumor suppressor genes. Forthcoming data from ongoing human trials will shed further light on the relationship between rAAV gene therapy and cancer.

## 4. Combination Therapy—rAAV and Chemotherapy

In addition to using rAAV vectors to treat multiple types of cancer, an orthogonal approach has been to combine rAAV gene therapy with currently available chemotherapeutics. Cytotoxic chemotherapies have been the clinical standard-of-care across a plethora of cancer types for decades. Although these cytotoxic chemotherapies can display effective responses, they are often associated with severe side-effects [70,71]. Furthermore, cytotoxic chemotherapies are not universally effective for all cancer types, and a subset of patients will experience the recurrence of drug-resistant tumors, at which point subsequent chemotherapies can be less effective [72,73,74]. Combining additional modes of therapies (also known as adjuvant therapies) with standard-of-care chemotherapy regimens could help minimize the side-effects associated with these regimens by lowering the required effective dose. In addition, enhanced tumor cell killing achieved with effective combinations could help to eliminate residual disease, thereby reducing the recurrence of drug-resistant tumors. rAAV-mediated transgene expression has been investigated as a potential avenue to selectively trigger cell death in tumor cells (as discussed extensively in the “Overview of Cancer Gene Therapy Approaches with rAAV” section), and in conjunction with cytotoxic chemotherapies, represents the potential for more effective combination therapy regimens.

Several pre-clinical studies have demonstrated that rAAV gene therapy in combination with cytotoxic chemotherapies enhances tumor cell killing in multiple tumor types including ovarian, gastric, colon, hepatocellular, and head and neck cancers (Table 2). In an orthotopic ovarian cancer mouse model, the combination of carboplatin, a standard-of-care chemotherapy in ovarian cancer, and rAAV-mediated delivery of the anti-angiogenic mutant endostatin (rAAV-P125A-endostatin) resulted in a significant decrease in tumor burden and an increase in survival compared to the single agents alone or the untreated group [75]. Although the mechanism for such an enhanced effect was not investigated, the authors noted that carboplatin treatment could sensitize the endothelial cells to P125A-endostatin, resulting in a further reduction of angiogenesis in ovarian tumors. Similarly, another study using ovarian cancer mouse models showed that treatment with the chemotherapy agents topotecan and paclitaxel resulted in increased survival of mice following the rAAV-mediated expression of bevacizumab, a monoclonal antibody directed towards VEGF, resulting in decreased angiogenesis [31]. In gastric cancer models, rAAV-mediated expression of the dominant negative survivin mutant Thr34Ala (rAAV-Sur-Mut(T34A)) resulted in decreased cell proliferation and increased apoptosis. Survivin, a member of the inhibitor of apoptosis (IAP) gene family, has been shown to be overexpressed in gastric cancer cells resulting in the inhibition of apoptosis. Overexpression of the dominant-negative survivin (T34A) abolished the anti-apoptotic effect exerted by the wild-type survivin. Importantly, combining 5-Fluorouracil (5-FU), the first-line chemotherapy drug for gastric cancer, resulted in enhanced tumor cell killing compared to single agents alone or the untreated group [76]. 5-FU treatment has been shown to induce survivin levels; therefore, the authors postulated that overexpression of the dominant-negative survivin with 5-FU treatment resulted in enhanced cytotoxicity [76]. Furthermore, enhanced tumor cell killing was demonstrated following the rAAV-mediated expression of survivin mutant T34A (rAAV-Sur-Mut(T34A)) and oxaliplatin, used for the treatment of advanced colorectal cancer that is resistant to 5-FU, in colon cancer models in vivo [77]. Yet another study showed that the combination of 5-FU and rAAV-mediated overexpression of shRNA targeting FHL2 (Four and a half LIM-only protein 2) resulted in increased colon cancer cell death in vivo [34]. Lastly, cisplatin, another chemotherapeutic used in clinics against multiple solid tumors, has been combined to elicit enhanced cell death with rAAV–TRAIL in hepatocellular carcinoma and head and neck squamous cell carcinoma in vivo [78,79]. TRAIL, a member of TNF-super family, has been previously shown to selectively trigger apoptosis in transformed cells.

The above studies clearly demonstrate that rAAV-mediated gene therapy could enhance clinical efficacy when combined with the standard-of-care chemotherapy; however, the mechanism associated with such enhanced efficacy following the combinations remains to be elucidated. It is well established that chemotherapy induces genotoxic and cytotoxic stress in tumor cells. Although each of these effective combinations could be acting through disparate mechanisms as discussed above, increased rAAV-mediated transduction following the increased stress response with chemotherapy may contribute to the enhanced cytotoxicity [22]. Interestingly, apart from paclitaxel, all of the aforementioned chemotherapies used in combination with rAAV vectors interfere with cellular processes occurring in S phase. Therefore, it is likely that an exacerbated stress response, which has been shown to enhance rAAV-mediated transgene expression, coupled with the chemotherapeutic drug’s interference in S phase progression, [80,81,82,83] could result in enhanced tumor cell death. This would allow for the combination of other chemotherapies and newer targeted therapies that induce a tumor-mediated stress response with rAAV-mediated gene therapy in multiple tumor types.

These studies should be pursued cautiously since similar mechanisms could also increase rAAV off-target tissue transduction resulting in increased rAAV-mediated toxicity; thus, approaches to limit rAAV-mediated transgene expression to tumor cells through optimizing the promoter, capsid, and/or delivery route should be explored. As previously noted, many of the synergistic effects of rAAV occurred with cytotoxic chemotherapies that target one or more cellular processes occurring in S phase; thus, elucidation of rAAV’s mechanistic interactions with these drugs in a cellular host context would likely lead to improved combination strategies for clinical applications. In summary, rAAV-mediated gene therapy could be explored as an avenue to enhance the efficacy and safety of the chemotherapeutic agents currently used in clinical settings.

## 5. Targeting Cancer Stem Cells with AAV

Cancer stem cells (CSCs) are a subpopulation of cancer cells residing within the solid tumor bulk (such as breast cancer or lung cancer) or hematological tumors (such as leukemia) [84] that have the potential for self-renewal and contribute to tumorigenesis and/or chemoresistance [85,86,87]. CSCs often participate in the epithelial–mesenchymal transition via the induction of an embryonic genetic program that re-expresses the genes SOX2, OCT4, NANOG, and DNMT1, and display stem cell-like profiles that contribute to tumor initiation, progression towards invasive phenotypes, therapeutic failure, and/or tumor recurrence [88]. Thus, it has been suggested that efficient tumor treatment requires eradication of the CSC population [89], which has generated considerable enthusiasm for the investigation of CSC-targeted therapies.

Gene therapy has long been proposed as a promising approach for cancer treatment [90], as illustrated in over 2144 clinical trials, accounting for 67.4% of all gene therapy clinical trials to date [90,91]. This technique could be applied to a wide range of tumor types, and a variety of genes and vectors are being used in clinical trials with successful outcomes [7]. Among these trials, 23 are currently underway to determine the efficacy of rAAV vectors for cancer therapeutics (Table 1). However, as summarized above, conventional strategies, such as enhancing the immune system to recognize the cancer cells, enhancing cancer cell apoptosis, and inducing anti-angiogenesis effects via regulation of the VEGF signaling pathway, are the major designs used in these cancer gene therapy clinical trials. Thus, alternative therapeutic approaches using AAV vectors to target CSCs would be a unique avenue of exploration.

One obvious strategy for CSC-targeted therapy is to target the CSC stemness features; this would require the use of AAV to mitigate the overexpression of the genes or perhaps miRs which play vital roles in maintaining the self-renewal capacity of CSCs. For example, one critical factor would be to identify an AAV capsid that could transduce the CSCs with high tropism and efficiency to confer CSC transduction specificity. Although the transduction efficiency of rAAV in different stem cells including embryonic stem cells (ESCs), hematopoietic stem cells (HSCs), and mesenchymal stem cells (MSCs) has been widely studied [92], to the best of our knowledge, there is no comparative transduction study of CSCs in vitro or in vivo. Although AAV6 capsids with site-specific modifications and CD34+ HSC-derived AAV variants are reported to have higher tropism to blood stem cells and support stable and efficient gene transfer [93], most other AAV serotypes may not productively transduce stem cells efficiently [94,95]. Unfortunately, cancer or CSC-derived AAV capsids have not yet been reported to date, consistent with reports of low to no germline transmission of AAV and an inverse relationship of wtAAV integrants in cancerous versus non-transformed cells. Indeed, Gao’s group [68] recently published a large-scale molecular epidemiological analysis of AAV in a cancer patient population, and found no significant difference in AAV prevalence, abundance, and variation between cancer and normal tissues. In addition, no specific AAV sequences predominated in tumor samples, and no clonality was observed [68]. This indicates a minimal chance that a natural CSC-derived AAV capsid exists and highlights an opportunity for directed evolution studies to isolate novel AAV capsid variants for increased CSC targeting.

In addition, previous reports have suggested that AAV vectors induce toxicity in some types of stem-like cells [59,96,97]. Johnston et al. reported that AAV ablates neurogenesis, and the ITR sequence alone is sufficient to induce cell death, suggesting AAV-linked toxicity in adult neural progenitor cells in mice [96,98]. Similarly, Hirsch et al. found that ESCs are particularly sensitive to AAV-induced cell death via a p53-dependent apoptotic response that is elicited by a telomeric sequence within the AAV ITR [59,92]. Reports of toxicity to other types of stem-like cells have shown mixed results, alluding to potentially differential effects of AAV toxicity depending on the species, type of stem cell, and AAV serotype and have been thoroughly discussed in a previous review [92,95,99,100,101,102]. When toxicity is observed, it is mostly thought to be induced through TLR activation via DNA sensors, especially the palindromic ITR hairpin DNA sequences [103]. These reports further indicate that optimization of the ITR sequences [2,104] (such as CpG depletion in the ITR structure) may be a useful strategy to counteract the potential toxicity, thus ensuring safer applications of rAAV that protect adult stem cell populations.

Notably, it is reported that ESCs and CSCs share some common biomarkers, gene signatures, signaling pathways, and epigenetic regulators [88]. This highlights the possibility that CSCs may also demonstrate toxicity in response to infection by wt or rAAV and illustrates the vast potential of AAV vectors (especially the ITR sequences) as a possible CSC-targeted therapy to supplement current cancer therapies and reduce tumor recurrence. However, before this exciting concept can progress to clinical translation in humans, attempts to better understand the CSCs in the context of AAV biology are needed. These approaches could include: (i) determining the wtAAV persistence in CSCs and the clinical consequences of its potential replication on CSCs and cancer cells, (ii) exploiting the ITR toxicity in CSCs, (iii) examining the potential interaction between the p53 transcriptional binding sites on AAV ITR sequences and CSC stemness, and (iv) the potential inhibitory effects of wtAAV on CSCs. Thus, a growing understanding of CSCs together with the favorable outcomes obtained from rAAV clinical trials [105] provides a common conceptual and research framework for basic and applied cancer research. Exploring AAV as vectors for CSC-targeted therapies would complement and expand the existing repertoire of therapeutic strategies for the treatment of cancer.

## 6. Closing Remarks

**Figure 2:**
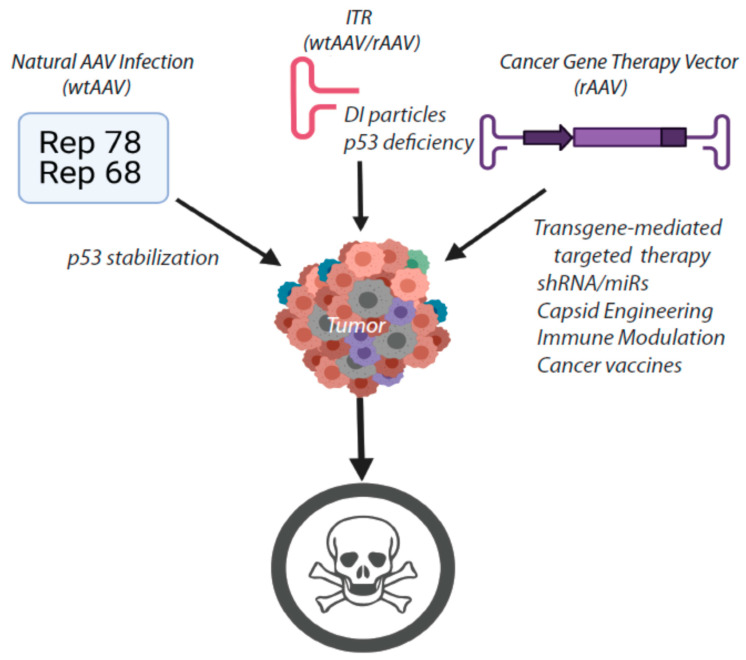
Summary of the potential attributes of AAV for cancer gene therapy.

In summary, AAV may be an excellent choice as an adjuvant therapy for multiple types of cancer. The genetic features of AAV combined with the cellular host response to AAV infection provide a unique opportunity to enhance the tumoricidal activity of cancer gene therapy vectors (Figure 2). Both the Rep protein(s) and the AAV ITRs exhibit independent tumoricidal activity in multiple contexts. Therefore, in combination with a transgene or other genetic components that specifically interfere with oncogenic signaling defects, AAV exhibits the potential to induce tumor regression and/or sustained remission, particularly when administered as an adjuvant therapy in conjunction with standard cytotoxic chemotherapies. Further mechanistic studies of AAV’s interactions with the cellular host machinery and the exploration of alternative transgenes/genetic cassettes would provide a basic scaffold for the development of novel approaches to treat cancer and lead to promising new avenues of therapeutic inquiry.

## Figures and Tables

**Table 1:** Clinical Trials Employing Viral-based Vectors for Cancer Gene Therapy Treatment [23]. Database Accessed on 6 March 2021.

Journal of Gene Medicine Database Trial ID	Start Date	Disease	Payload	Phase
CH-0025	2001	Malignant Melanoma	GM-CSF B7.2	1
CN-0020	2008	Malignant Solid Tumors	Tumor Antigen	1
CN-0028	2012	Gastric Cancer	Carcinoembryonic antigen (CEA)	1
ES-0021	2012	Pancreatic Cancer	Hyaluronidase	1
JP-0014	N/A	Hormone refractory metastatic prostate cancer	HSV-TK	1
NL-0012	2004	Hormone refractory prostate cancer	GM-CSF	1
NL-0013	2006	Metastatic Prostate Cancer	GM-CSF	3
NL-0014	2006	Prostate Cancer	GM-CSF	3
NL-0015	2006	Prostate Cancer	GM-CSF	3
NL-0016	N/A	Prostate Cancer	GM-CSF	1
NL-0021	2005	Prostate Cancer	IL-12	1
UK-0133	2005	Prostate Cancer	GM-CSF	3
UK-0134	2005	Prostate Cancer	GM-CSF	3
US-0459	2001	Hormone-Refractory Prostate Cancer	GM-CSF	1
US-0493	2001	Hormone Refractory Prostate Cancer	GM-CSF	1/2
US-0653	2004	Hormone-Refractory Prostate Cancer	GM-CSF	3
US-0675	2004	Prostate Cancer	GM-CSF	1/2
US-0708	2005	Prostate Cancer	GM-CSF	3
US-0903	2008	Prostate Cancer	GM-CSF	2
US-1165	2012	Prostate Cancer	GM-CSF	1/2
US-1748	2018	Non-Hodgkin’s Lymphoma/B-cell Acute Lymphoblastic Leukemia	CD19, CD8a, N6 and TCRζ	1
US-1800	2018	Multiple Myeloma	CAR2-α-BCMA, CD28/CD3ζ	1

**Table 2:** Preclinical studies conducted using rAAV and chemotherapy combinations.

Cancer Type	Chemotherapy	Transgene	rAAV Capsid, Promoter	References
Ovarian	Carboplatin	Endostatin (P125A)	Unknown, CGA	[75]
Ovarian	Topotecan and Paclitaxel	Bevacizumab Ab	rh.10, CGA	[31]
Gastric	5-FU	Survivin (T34A)	Unknown, CGA	[76]
Colorectal	Oxaliplatin	Survivin (T34A)	Unknown, CGA	[77]
Colorectal	5-FU	shRNA FHL2	AAV2, U6	[34]
Hepatocellular	Cisplatin	TRAIL	AAV2, hTERT	[78]
Head and Neck	Cisplatin	TRAIL	Unknown, CGA	[79]

Notes: CGA = CMV enhancer, chicken beta-Actin promoter; hTERT = human reverse transcriptase component; 5-FU = 5-Fluorouracil.
